# Cost analysis of hospitalized Clostridium difficile-associated diarrhea (CDAD)

**DOI:** 10.3205/dgkh000256

**Published:** 2015-10-29

**Authors:** Claudia Hübner, Nils-Olaf Hübner, Michaela Muhr, Franziska Claus, Henning Leesch, Axel Kramer, Steffen Flessa

**Affiliations:** 1 Department of Health Care Management, University of Greifswald, Greifswald, Germany; 2 Institute of Hygiene and Environmental Medicine, University Medicine Greifswald, Greifswald, Germany; 3 Department of Economics and Financial Management, University of Greifswald, Greifswald, Germany

**Keywords:** Clostridium difficile, nosocomial infections, opportunity costs, extension of length of stay

## Abstract

**Aim:** *Clostridium difficile*-associated diarrhea (CDAD) causes heavy financial burden on healthcare systems worldwide. As with all hospital-acquired infections, prolonged hospital stays are the main cost driver. Previous cost studies only include hospital billing data and compare the length of stay in contrast to non-infected patients. To date, a survey of actual cost has not yet been conducted.

**Method:** A retrospective analysis of data for patients with nosocomial CDAD was carried out over a 1-year period at the University Hospital of Greifswald. Based on identification of CDAD related treatment processes, cost of hygienic measures, antibiotics and laboratory as well as revenue losses due to bed blockage and increased length of stay were calculated.

**Results:** 19 patients were included in the analysis. On average, a CDAD patient causes additional costs of € 5,262.96. Revenue losses due to extended length of stay take the highest proportion with € 2,555.59 per case, followed by loss in revenue due to bed blockage during isolation with € 2,413.08 per case. Overall, these opportunity costs accounted for 94.41% of total costs. In contrast, costs for hygienic measures (€ 253.98), pharmaceuticals (€ 22.88) and laboratory (€ 17.44) are quite low.

**Conclusion:** CDAD results in significant additional costs for the hospital. This survey of actual costs confirms previous study results.

## Zusammenfassung

**Zielsetzung:** Die *Clostridium-difficile*-assoziierte Diarrhö (CDAD) bedingt hohe finanzielle Belastungen für Gesundheitssysteme weltweit. Wie bei allen nosokomial erworbenen Infektionen ist ein verlängerter Krankenhausaufenthalt der wesentliche Kostentreiber. Bisherige Kostenstudien beziehen sich nur auf Krankenhausabrechnungsdaten vor Einführung des DRG-Entgeltsystems und den Vergleich von Verweildauer zu nicht infizierten Patienten. Eine Erhebung tatsächlicher Kosten steht bislang aus.

**Methode:** Anhand einer retrospektiven Analyse wurden Daten der Universitätsmedizin Greifswald von Patienten mit einer stationär behandelten CDAD über einen 1-Jahres-Zeitraum ausgewertet. Über eine Identifizierung von CDAD-relevanten Behandlungsprozessen wurden die Kosten von Hygienemaßnahmen, Arzneimittel und Labor sowie Erlösausfälle bedingt durch Bettensperrungen und Verweildauerverlängerungen berechnet.

**Ergebnisse: **19 Patienten wurden in die Analyse eingeschlossen. Im Durchschnitt fallen pro CDADPatient zusätzliche Gesamtkosten in Höhe von 5.262,96 € an. Erlösausfälle aufgrund der verlängerten Verweildauer stellen mit 2.555,59 € pro Fall den höchsten Anteil dar, gefolgt von den Erlösausfällen aufgrund von Bettensperrungen während der Isolierung mit 2.413,08 € pro Fall. Insgesamt ergeben diese Opportunitätskosten einen Anteil von 94,41% an den Gesamtkosten. Die Kosten für Hygienemaßnahmen (253,98 €), Arzneimittel (22,88 €) und Labor (17,44 €) sind dem gegenüber gering.

**Schlussfolgerung:** Die CDAD führt zu deutlichen Mehrkosten für das Krankenhaus. Unsere Erhebung der tatsächlichen Kosten bestätigt bisherige Studienergebnisse.

## Introduction

*Clostridium difficile*-associated diarrhea (CDAD) is the main cause of infectious diarrhea in hospitalized patients [1]. This results in huge financial burden on health care systems worldwide. The costs due to CDAD infections are estimated at over 3 billion euros per year for Europe alone [2]. In the US the costs are estimated at more than 1.1 billion US dollars per year [3]. It can be assumed that the costs will nearly double over the next 4 decades due to an aging population [2]. As with all nosocomial infections prolonged hospitalization is the major cost driver. Patients occupy the limited capacity of beds and require additional diagnostic and therapeutic interventions [4].

In literature, some studies carrying out a cost impact assessment in the treatment of CDAD patients are evident [5], [6], [7]. However, these studies are based on an analysis of hospital billing data and a comparison in length of stay to non-infected patients. Therefore, actual costs caused by *Clostridium difficile* infections from a hospital perspective are not reflected. For the first time McGlone et al. examined the economic importance using a mathematical model as well as a scenario analysis [8]. However, this work is exclusively based on theoretical assumptions referencing to data taken from literature. Own patient data was not included.

The present study determines the actual cost of CDAD in a hospital setting. For this purpose, those sub-processes of treatment unambiguously and exclusively related to CDAD are identified and the respective costs are calculated. The costs of hygienic measures, pharmaceuticals, laboratory costs and losses in hospital revenue due to the blocking of beds during isolation as well as an extended length of stay will be addressed in particular.

## Methods

### Setting and design of study

Data of the Greifswald University Hospital, an academic teaching hospital as well as a tertiary care facility with 880 beds, was used for the analysis. In 2010 35,324 inpatient cases were treated here.

A retrospective analysis of patients suffering from CDAD during hospitalization was performed. Microbiological findings as well as data taken from the medical controlling of the Greifswald University Hospital over a period of one year formed the basis of the data collection.

### Inclusion and exclusion criteria for patient data

Initially all patients treated at the Greifswald University Hospital in 2010 showing an additional diagnosis of enterocolitis due to clostridium difficile (ICD-10 code A04.7) and positive microbiological findings (toxin detection) were identified for the analysis. Subsequently, the data was adjusted for those patients exhibiting an additional MRSA infection, being artificially ventilated or meeting both criteria. In those cases the costs due to CDAD could not clearly be separated from the costs of additional factors. Therefore, these patients were excluded. Availability of complete medical records was necessary in analyzing the costs of pharmaceuticals and isolation.

### Cost calculation

#### Hygienic costs

The determination of processes occurring in hygiene management was carried out based on standard in-house operating instructions for CDAD. A classification of sub-processes taking place on the first day, the following days and the last day of isolation was performed. The implementation of processes is very similar to those of the in-house management of MRSA cases. Therefore, the periods according to the MRSA documentation form for the proof of the OPS 8.987 (classification of operational procedures; German DRG system) encoding could be taken as the basis for calculating personnel expenses (Table 1 (Tab. 1)). The times were multiplied with a price per minute of € 0.299 determined from the average gross wage of a full-time nurse (plus employer contribution, according to the German collective agreement of the civil service applying to hospitals *TVöD-K*).

The expenses for material during isolation consist of coats, gloves and disinfectants used. The average quantities were directly requested in the wards and offset against the purchasing prices of the hospital.

#### Pharmaceutical costs

In the present work, only additionally administered antibiotics due to a clostridium difficile infection (Metronidazol or Vancomcyn) were taken into account in calculating pharmaceutical costs. For this purpose, individual patient records were reviewed. The type of antibiotic, quantity and period of administration was recorded for the respective patient. These were valued at purchase prices of the University Hospital Pharmacy.

#### Laboratory costs

The calculation of costs of diagnosis was based on the price information taken from the German uniform assessment standard (EBM) for medical services [9]. According to the EBM billing item 32 707 € 11.90 were estimated for a toxin screening test. The costs of creating a culture was calculated at € 6.40 in case of a negative test result (according to EBM billing item 32 726). € 3.10 were charged additionally if due to a positive test result the Gram stain method had to be applied.

#### Opportunity costs

Opportunity costs are defined as loss of revenue for the hospital, which arise when due to an occupied or blocked bed no new case can be treated. This is based on the realistic assumption that the hospital is working at full capacity and new patients would have to be rejected. Variable costs reduce the amount of revenue loss since the hospitals saves costs by not treating a new case [4], [10]. In literature, the proportion of variable costs in total costs is indicated at about 25% [11].

These costs incurred in the treatment of CDAD patients for one due to a blockage of beds during isolation and for another due to an increase in the total length of stay compared to non-infected patients.

**Figure 1:**
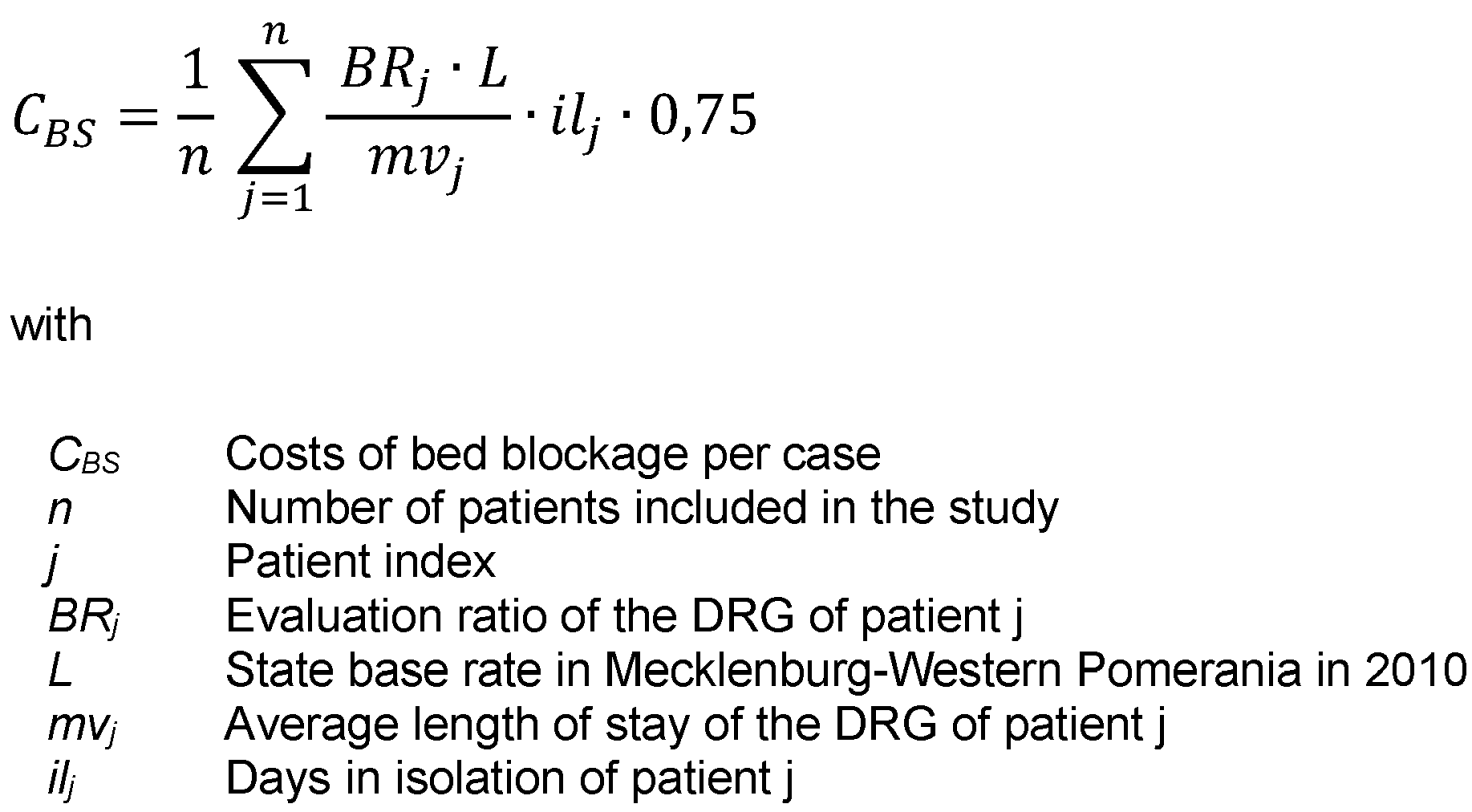
Costs of bed blockage per case

The costs caused by blocking beds during isolation result from the average revenue a hospital generates from the occupancy of a bed and the duration of isolation. In the calculation, the average revenue was generated based on the G-DRG lump sum remuneration catalogue (2010 version) over all DRGs per day of patients included in the study, as there was no data available on average values over all inpatient cases treated in 2010 [12]. In addition, it was assumed that on average patients are accommodated in a double room. Consequentially, an extra bed is blocked during the period of isolation. This was with regard to the recommendation of the Robert Koch Institute on the accommodation of CDAD patients in an individual room [13]. The share of variable costs amounting to 25% is being deducted (Figure 1 (Fig. 1)).

**Figure 2:**
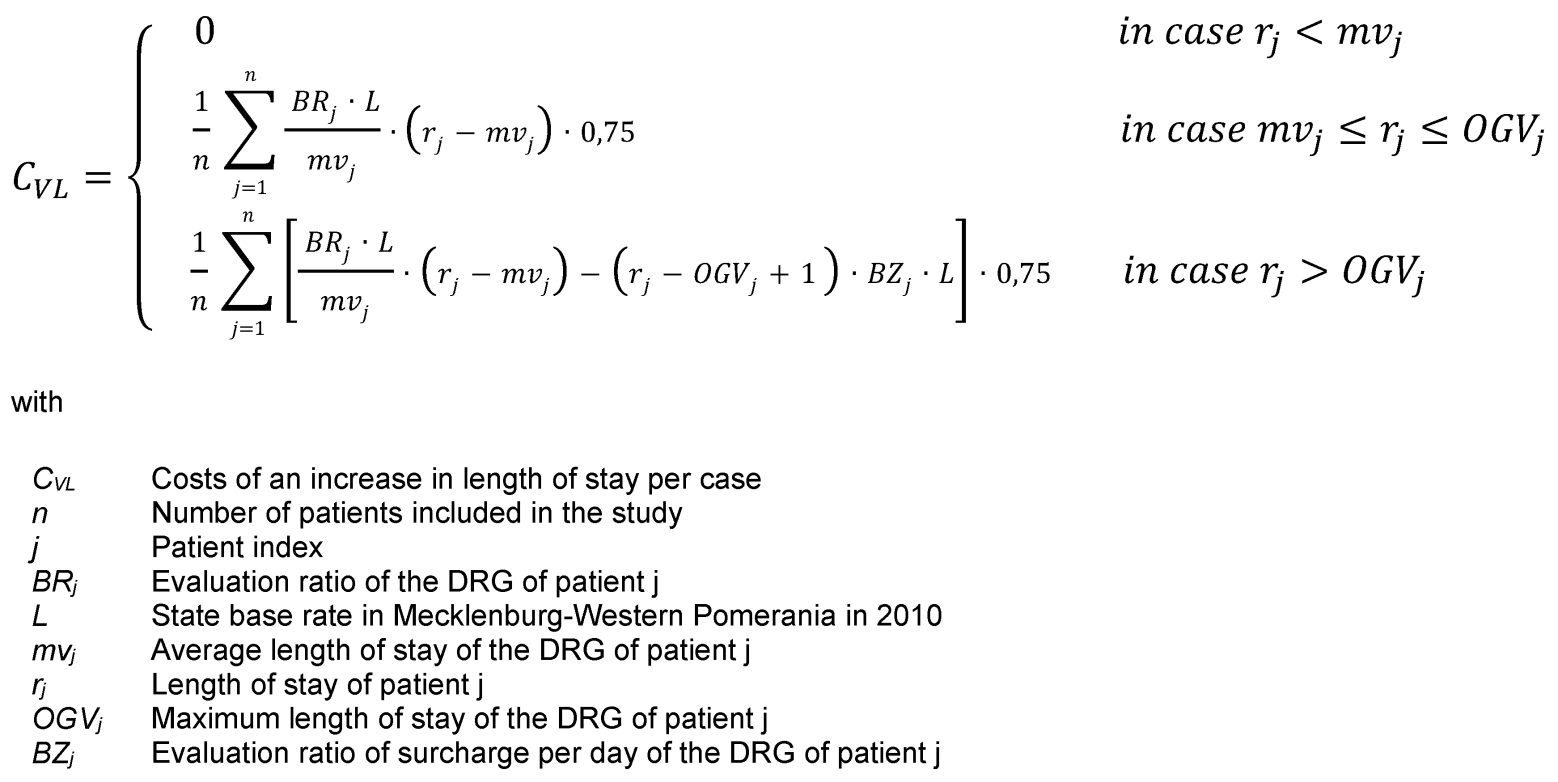
Costs of an increase in length of stay per case

The costs resulting from an increase in length of stay in CDAD patients were also determined based on the G-DRG lump sum remuneration catalogue [12]. These resulted from a multiplication of average revenue per bed per day with the difference between actual length of stay and average length of stay minus any surcharge received in case of an increase exceeding the maximum length of stay. There are two possible variations of the formula, depending on whether this surcharge due to exceeding the maximum length of stay arose. Opportunity costs were set at a value of 0 in the event of the actual length of stay falling below the average length of stay (Figure 2 (Fig. 2)).

## Results

### Patient data

A total of 43 patients were identified showing an additional diagnosis of CDAD as well as a positive screening for toxin. 27 patients remained after the adjustment of the data for cases of MRSA and/or artificial ventilation. In 8 more patients the graphs could not be determined based on the patient’s record. Thus, calculations could not be performed in those cases.

Consequentially, 19 patients were included in the analysis. 10 out of 19 patients were male (52.6%) and 9 were female (47.4%). An average age of 75.4 years (29–91) was observed. The average age in women at 73.7 years was lower than those in men at 77 years.

Overall, 16 out of 19 patients showed a nosocomial infection with Clostridium difficile (84.2%). The length of hospital stay ranged between 5 and 60 days. This equals an average of 23.3 days. The mean value of days spent in isolation was at 7.8 days (range: 2–20).

### Types of costs

#### Hygienic costs

An overview of costs for hygiene measures is presented in Table 2 (Tab. 2) and Table 3 (Tab. 3). On average, a total of € 253.99 per case was determined consisting of € 150.95 for personnel expenses and € 103.04 for material expenses. 

#### Pharmaceutical costs

The average costs of medication per day of therapy were at € 3.34. In 13 patients, a monotherapy with metronidazole (1 to 3 times daily 500 mg, equals € 0.49–1.47 per day) was administered. Vancomycin only was administered in 2 more patients (3 times daily 500 mg at € 4.35 per day or 2 times daily 1,000 mg at € 5.88 per day respectively). In the remaining 4 patients metronidazole and vancomycin were partly combined over the period of treatment. In those cases, the costs of therapy per day ranged from € 2.92 to € 9.94. With an average duration of treatment of 6.8 days, the average costs per case amounted to € 22.88.

#### Laboratory costs

In diagnostic costs, the expenses for a toxin screening test were considered at € 11.90 for each patient. Additionally, a culture was grown for 15 patients. In case of a negative result (n=12) costs in the amount of € 6.40 incurred, in case of a positive result (n=3) in the amount of € 9.50. Thus, the average laboratory costs per case amounted to € 17.44.

#### Opportunity costs

An on average loss in revenue per day of € 307.71 was determined for a blocked bed. For an average period in isolation of 7.8 days a loss in revenue of € 2,413.08 per case due to bed blockage was determined. On average, patients with CDAD stayed 11.4 more days in hospital compared to the average length of stay according to the German DRG payment system catalogue. The overall loss in revenue due to an increased length of stay amounted to € 2,555.59.

#### Total costs

The calculated total costs for all 19 patients are summarized in Table 4 (Tab. 4). On average, total additional costs in the amount of € 5,262.96 incurred per CDAD patient.

**Figure 3:**
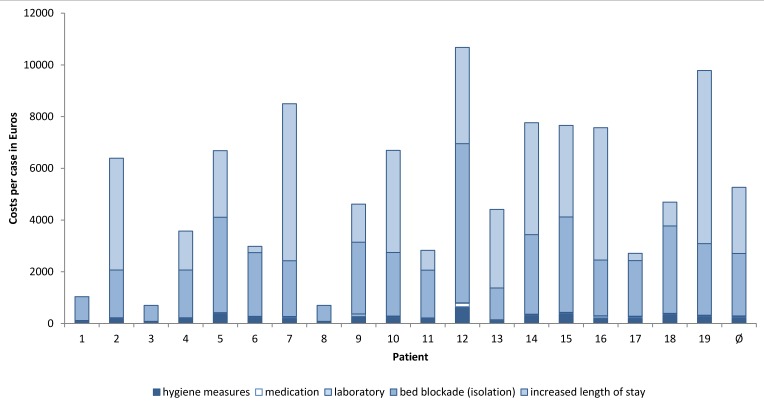
Distribution of costs for hospitalized CDAD patients

Figure 3 (Fig. 3) displays the individual cost elements per case at the respective amount. On average, the loss in revenue due to an extended length of stay accounted for the highest share (48.56%) followed by revenue loss due to bed blockage during isolation of CDAD patients (45.85%). Overall, the opportunity costs make up for 94.41% of total costs. The expenses for hygiene measures (4.83%), pharmaceuticals (0.43%) and laboratory (0.33%) are comparably low.

## Discussion

With the present work, a differentiated analysis of the additional costs of *Clostridium difficile*-associated diarrhea in hospitalized patients was conducted. Unlike previously published studies, this analysis is not only based on revenue data but also includes the assessed processes of hygiene management, pharmaceutical and laboratory expenses as well as the opportunity costs arising from revenue losses for the hospital.

The length of stay for CDAD patients in hospitals as reported in literature varies from 16 to 37 days [14]. Therefore, the determined average length of stay is 23.3 days within this interval. An increase in length of stay of 11.4 days also fits in with the previously published results of other studies. The closest approach to the result is provided by the study of Al-Eidan et al. determining an increased length of stay of 13 days in comparison to non-infected patients [5]. Vonberg et al. and Dubberke et al. show lower values of 17 and 6 days respectively [6], [15], Wilcox et al. conclude a prolonged period of hospitalization of 21.3 days [7].

Within their review, Wiegand et al. refer to 3 European studies analyzing the costs of Clostridium difficile infection [14]. In their 2006 study at the Medical School of Hannover, Vonberg et al. show additional costs of € 7,147 per CDAD patient [6]. The present analysis determined lower total additional costs per case at € 5,262.96. Both Al-Eidan et al. and Wilcox et al. refer to older data of the years 1994–1995 [5], [7] Taking into account the price index of health services, as projected for 2010, similar values are obtained with £ 6,986 (€ 8,645) and £ 4,577 (€ 5,664) respectively [14].

Within this analysis, the opportunity costs (94.41%; € 4,968.67) account for the largest component. For the first time, these were analyzed based on DRG billing data. Earlier studies include the previously applicable remuneration rates per inpatient day in calculating the losses in revenue per day. A similar proportion was determined by Al-Eidan et al. at 93.8% as well as by Wilcox at 94% [5], [7]. McGlone et al. confirm these results. Costs solely due to an increase in length of stay of 6 to 14 days are indicated between $ 9,179 (€ 6,728) and $ 11,456 (€ 8,397) [8].

Various studies prove the treatment with metronidazole cheaper in comparison to vancomycin [1]. In their 1998 study, Butterworth et al. calculated costs in the amount of $ 3.37 (€ 3.12) for a 10 day treatment with metronidazole, while a 10 day vancomycin therapy led to costs of $ 616.71 (€ 571.36) [16]. With Al Eidan et al. the differences were less significant. For an average period of therapy of 7.5 days the costs of metronidazole amounted to £ 1.60 (€ 1.99) and the ones for vancomycin to £ 162.50 (€ 201) [5]. A difference in price between these two approaches to therapy could also be detected within the present observations. It was found that the recommended duration of therapy of 10 days has largely not been complied with. An average of 6.8 days was determined. Possible underlying reasons were the decease of patients as well as the transfer to other hospitals.

The study shows limitations that should be considered in the assessment. Since the analysis is a retrospective one, all advantages and disadvantages with regard to data quality, the amount of included patients as well as the application to a real, non-study situation have to be acknowledged. In considering the results the relatively small study population should be addressed. Comparison to other studies provides no evidence of specifics in the sample limiting the comparability.

The analysis of laboratory costs was based on the German uniform assessment standard (EBM) for physician services. This does not reflect the actual costs incurred. Still, an orientation is provided especially since the internal accounting of the University Hospital has so far been carried out on the basis of the EBM standard. An additional limitation of this study is with regard to hygienic costs. More (patient-remote) processes occur that were not considered in the analysis. The extent to which this affects the overall cost of treatment cannot be estimated and could be examined in further observations. In addition, expenses incurred in hospital hygiene, i.e. for on-site consultation and the registration of cases. Therefore, the determined costs represent a resilient lower limit of hygiene expenses.

The calculation of the opportunity costs is based on the assumption that the hospitals are working at full capacity. Only then would each blocked bed be used by another patient in the same period. Since this distribution can differ from hospital to hospital as well as throughout the year, this would have to be differentiated for the individual case study. Furthermore, an on average accommodation of isolated patients in a double room was assumed. Depending on the respective facilities and the capacity utilization a hospital will seek to first use available single rooms for isolation in order to avoid revenue loss. On the other hand, especially older facilities are predominantly equipped with multi-bed rooms accommodating 3 to 4 patients per room. Moreover, alongside a single room accommodation cohort isolation in several simultaneously occurring cases is possible [13]. The analysis therefore attempted to replicate an average. Furthermore, the average length of stay corresponding to the respective DRG of treatment was applied in calculating the deviation in length of stay. However, in order to maximize the contribution margin a hospital would tend to keep the day of discharge close to the minimum length of stay. As a result, the opportunity costs were even higher due to the increase in length of stay.

To summarize, it can be stated that CDAD leads to significant additional costs for the hospital. Consistency in hygiene with standard processes is recommended in order to avoid transmission to other patients as well as to treat infected patients efficiently. In this way, the period of isolation and of hospitalization can be as short as possible.

## Notes

### Second publication

This article is a translation of the original publication: Hübner C, Hübner NO, Muhr M, Claus F, Leesch H, Kramer A, Fleßa S. Kostenanalyse der stationär behandelten Clostridium-difficile-assoziierten Diarrhö (CDAD). Gesundh ökon Qual manag. 2013;18(2):80-5. DOI: 10.1055/s-0032-1330635

### Statement of the authors

The authors declare not having any financial ties to a company whose product has an important part within the article (or a company selling a competing product).

The study is part of the project network “HICARE – Health Region Baltic Sea Coast, Action Alliance against Multi-resistant Bacteria” funded through the Federal Ministry of Education and Research and the state of Mecklenburg-Western Pomerania.

## Figures and Tables

**Table 1:**
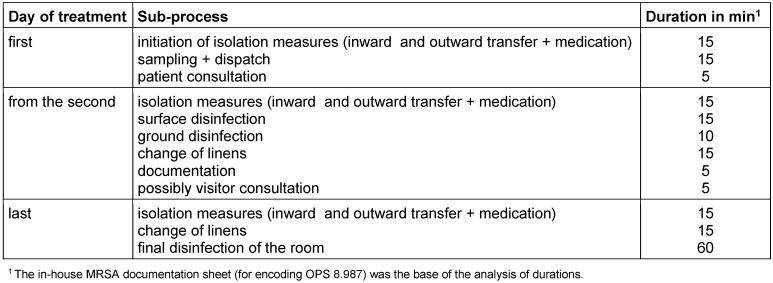
Sub-processes of hygiene management, average duration of activity

**Table 2:**
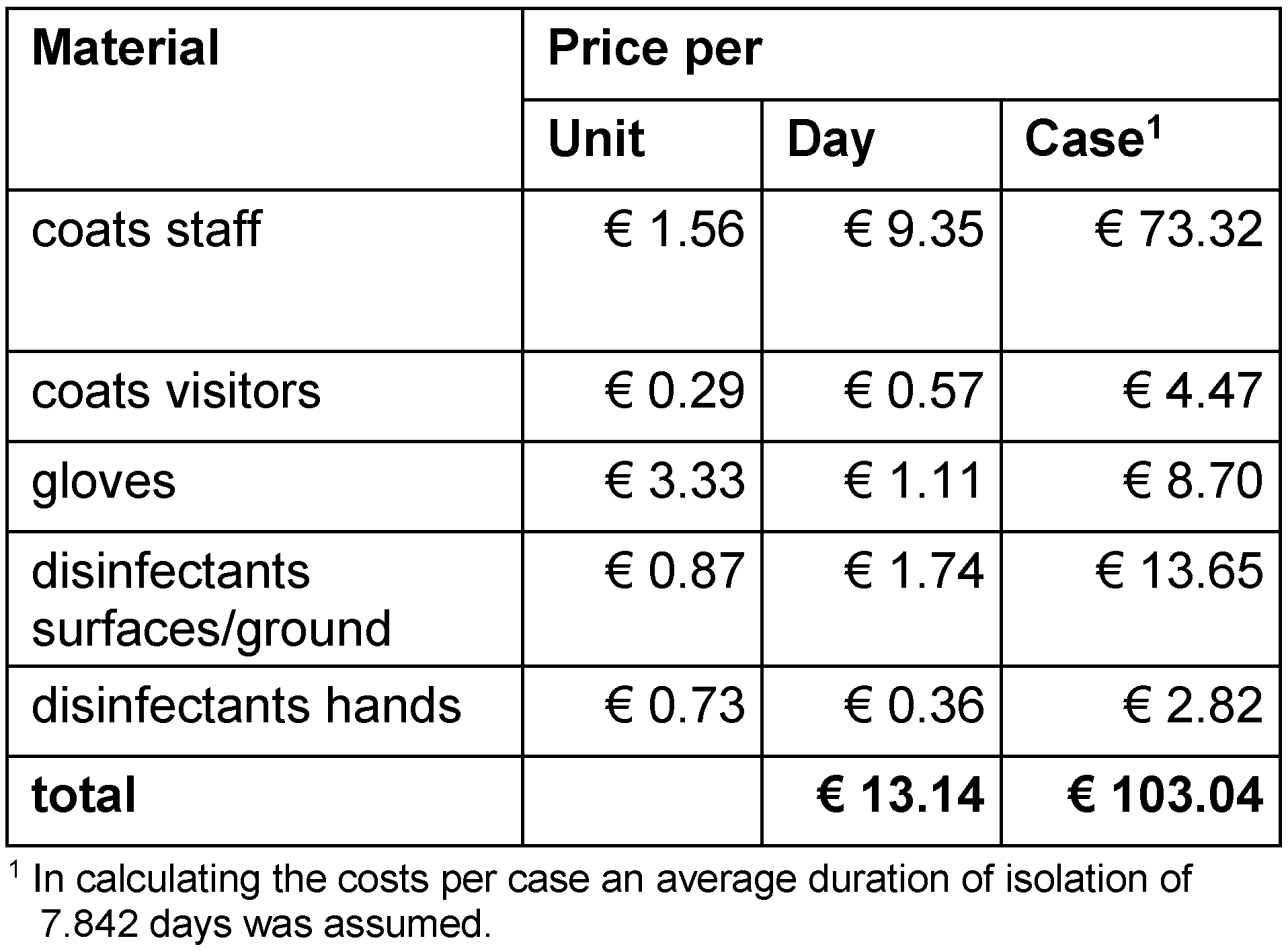
Material costs of hygiene measures

**Table 3:**
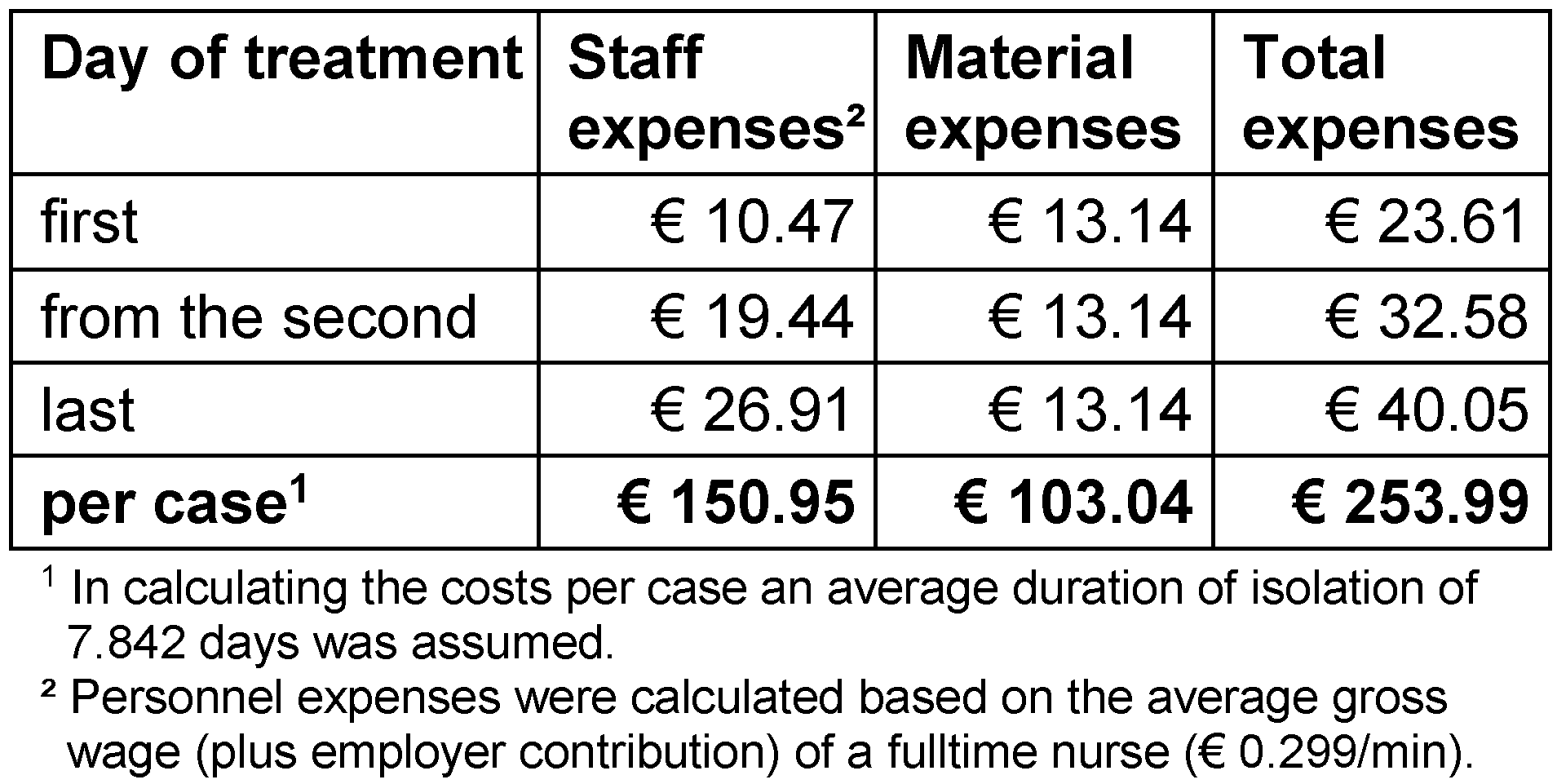
Costs of hygiene management (excluding revenue losses during isolation)

**Table 4:**
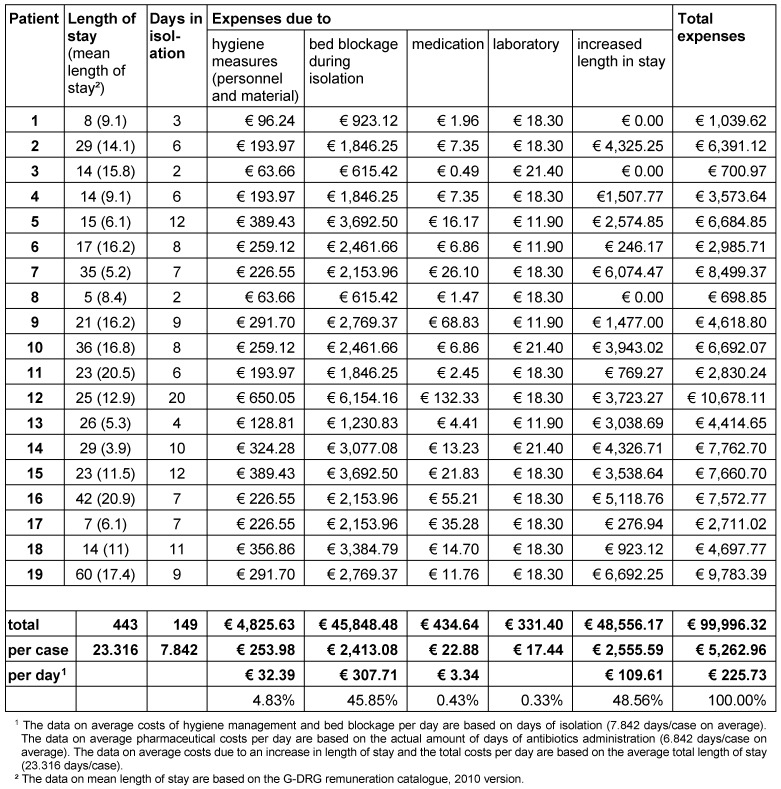
Allocation of total additional costs of CDAD patients
